# The Development of a Communication Tool to Aid Parent-Centered Communication between Parents and Healthcare Professionals: A Quality Improvement Project

**DOI:** 10.3390/healthcare11202706

**Published:** 2023-10-10

**Authors:** Luise V. Marino, Nicole Collaḉo, Sophie Coyne, Megan Leppan, Steve Ridgeway, Tara Bharucha, Colette Cochrane, Catarina Fandinga, Karla Palframan, Leanne Rees, Ahmed Osman, Mark J. Johnson, Anna Hurley-Wallace, Anne-Sophie E. Darlington

**Affiliations:** 1 Paediatric Intensive Care, Southampton Children’s Hospital, University Southampton Hospital NHS Foundation Trust, Southampton SO16 6YD, UK; ahmed.osman@uhs.nhs.uk; 2 School of Health Sciences, Southampton University, Southampton SO17 1BJ, UK; n.b.collaco@uhs.nhs.uk (N.C.);; 3 Raised By Wolves, London W1F 7PR, UK; sophie@rbw.london (S.C.); steve@rbw.london (S.R.); 4 Paediatric Cardiology, Southampton Children’s Hospital, University Southampton Hospital NHS Foundation Trust, Southampton SO16 6YD, UK; tara.bharucha@uhs.nhs.uk (T.B.); colette.cochran@uhs.nhs.uk (C.C.); 5 Department of Dietetics/Speech Language Therapy, University Southampton Hospital NHS Foundation Trust, Southampton SO16 6YD, UK; catarina.fandinga@uhs.nhs.uk (C.F.); karla.palframan@uhs.nhs.uk (K.P.); leanne.rees@uhs.nhs.uk (L.R.); 6 Neonatal Medicine, Princess Anne Hospital, University Southampton Hospital NHS Foundation Trust, Southampton SO16 6YD, UK; m.johnson@soton.ac.uk

**Keywords:** complex medical needs, family centered care, shared decision making, children, communication

## Abstract

Good communication is central to good healthcare. As a result of poor communication between parents and healthcare professionals (HCPs) in clinical settings, this study aimed to address this problem by developing a communication tool to empower parents and act as a prompt for HCPs to talk about the child’s care and gather information at the point of admission to hospital about what is important to families, therefore supporting patient-centered communication. A design thinking process was used to develop a physical copy of Chloe’s card and evaluate its use. Design thinking is a problem-solving approach, which uses an empathetic lens to integrate viewpoints of different stakeholders throughout the process of creating solutions. Design thinking involves five processes: (1) empathise—including a literature review and data synthesis, (2) define—by completing semi-structured interviews with parents about their experience of communication and HCPs perceptions of parent’s experience of communication, (3) ideate—iterate the design of Chloe’s card with parents and HCPs, (4) prototype—develop the design of Chloe’s card, and (5) test—pilot test in clinical practice. Results from this initial study suggest that a small hand-held card, with emoticons and a place to write concerns, was acceptable to parents and feasible to use in clinical practice. Parents do not always feel heard by HCPs and a tool such as Chloe’s card may help facilitate sharing of information about matters important to them and their child. However, some HCPs felt the need for a communication tool undermined their clinical skills. Feedback from HCP participants suggests that the idea of Chloe’s card was acceptable and perceived as potentially being useful in clinical practice. Further work is required, as part of a larger study, to further refine this communication tool, identify those parents who would benefit most from Chloe’s card, as well as to further refine the HCP process prior to implementing it into clinical settings. It was noted future iterations would benefit from a digital version linked with a child’s electronic record, as well as multi-language versions and information for parents.

## 1. Introduction

Good communication is a central part of high-quality care [1]. Ineffective communication between healthcare professionals (HCPs), patients, and families can lead to poor experiences of care [2], and in extreme cases, litigation [3]. The most common complaints arising from healthcare users in the United Kingdom (UK) are as a result of communication breakdown [4], leading to patient dissatisfaction [5]. Children’s hospitals often provide complex care to a wide range of families and children, all of which require collaboration from a well-functioning interdisciplinary team. However, the Care Quality Commission in the UK reports that although many children and parents have a positive experience of healthcare, 15% of children do not feel listened to or involved in decisions around their care, and 30% of healthcare professionals failed to communicate with families in a way they could understand [6]. Medical advances mean more children are likely to survive a serious illness, but may face a range of complex medical, physical, nutritional, and psychosocial consequences requiring access to various healthcare environments [7]. For example, parents of children with congenital heart disease (CHD) often experience stress, depression, and anxiety during [8] and following hospitalisation [9], that may have an impact on parental participation in decision making [10,11].

Children with medical complexity may have periods of illness where medical facts are often presented with a range of treatment options [12]. Shared care models are developed in adult medicine, and can be defined as ‘a partnership between health care providers and patients, in which each contributes equally to decisions about different aspects of treatment’ [13]. Shared care models are increasingly used in the care of children, but often involve HCPs completing checklists of task-orientated healthcare transactions as part of patient communication and safety care bundles [14]. There is a paucity of evidence supporting how these models work to improve communication and outcomes in children [15,16] and few HCPs received any formal training as how to deliver this type of care [13,17]. In addition, systematic and meta-analysis reviews report that the effectiveness of share care models is low [7,18,19], and due to a lack of standardisation, parental experience is not always improved and distress arises from conflicting opinions of healthcare professionals [20]. Recent work by Jacobs et al. [12] suggests that more evidence is required around the development and implementation of communication tools in children with medical complexity to reduce power imbalances and improve care as well as the availability and access to information resources.

Although it is not feasible to develop decision aids for shared decision making for all of the choices available to parents of children with medical complexity, families may benefit from communication tools [17]. Towle et al. [21] described eight principles that HCPs should adopt in order to practice patient-centered care based on shared decision making, which include developing a partnership with the patients (parents), reviewing the parents’ preference for information, reviewing the parents preference for their role in decision making, responding to parents ideas, concerns, and expectations, identifying choices, presenting evidence, facilitating a decision, and agreeing on an action plan. However, Nakagawa et al. [22] propose additional subtleties to the shared care model by focusing on family values through more specific follow-up questions such as ‘tell me more’, ‘why’, and ‘what else’. This helps HCPs identify values and priorities [22]. We previously sought to improve clinical and growth outcomes of infants with CHD [23,24,25] using principles of shared decision making around feeding choices. Recommendations for national service improvements by Brown et al. identified a number of priority areas, including the development of shared decision making and communication tools, training, escalation of concerns, and information for both families and HCPs [9].

For parents to be able to cope with challenging situations, HCPs need to be able to listen to parents, answers questions, and provide emotional support [26]. Effective communication, as part of shared decision making between HCPs and parents, is important not only for parental satisfaction, but also for the sake of HCPs’ own well-being. Studies suggest that HCPs lacking confidence in their own communication skills and difficulties in communication may lead to increased stress and contribute to staff burnout [27,28]. Recommendations for future research around communication interventions suggest that considerations should be given to the transferability of resource, accessibility, and awareness of tools along with the cost implication of an intervention, particularly staff resources [29,30,31,32]. Therefore, this study aimed to develop a parent-held communication tool and test it in practice. In this article, we describe the phases of work completed to understand the problem (features of communication to improve), design a potential solution, and explore how this worked in a clinical setting. The questions guiding our quality improvement project using design thinking [29] were:

1. How can we support parents to feel empowered to communicate their values and priorities to HCPs?
2. What format should a communication tool take?
3. How does the communication tool work in clinical practice?

## 2. Materials and Methods

### 2.1. Setting

University Hospital Southampton NHS Foundation Trust (UHS) is home to the Southampton Children’s Hospital, a secondary and tertiary center providing paediatric services and acute specialist care for children locally and across the South of England and the Channel Islands.

### 2.2. Developing and Designing the Intervention

**Figure 1:**
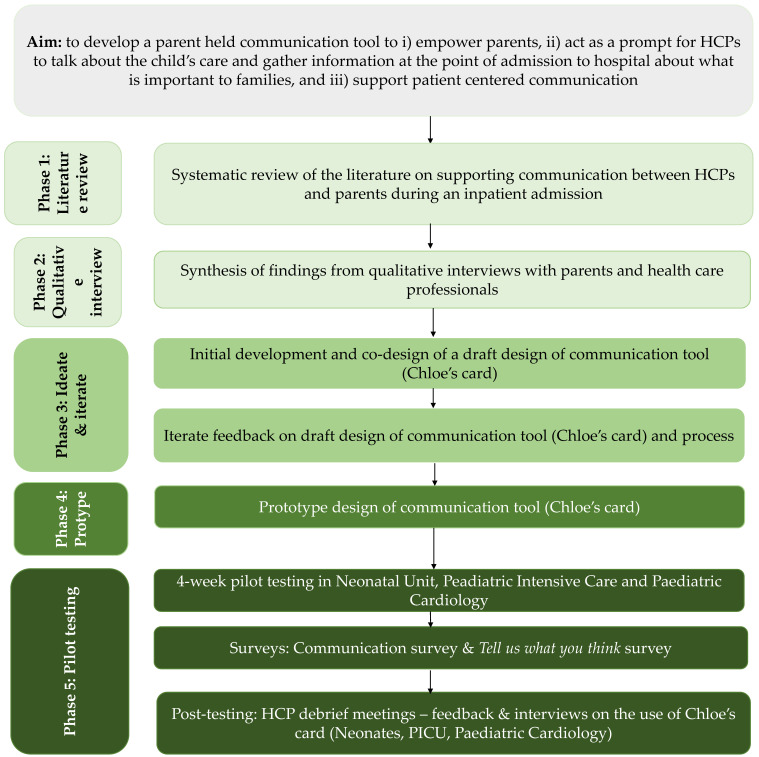
Intervention development and pilot testing.

A design thinking process was used to develop a communication intervention tool to support shared decision making. Design thinking is a problem-solving approach that uses an empathetic lens to integrate viewpoints of different stakeholders throughout the process of creating solutions [29,30]. Design thinking involves five phases: (1) empathise—including a literature review and data synthesis, (2) define—by completing semi-structured interviews with parents about their experience of communication and HCPs perceptions of parent’s experience of communication, (3) ideate—iterate the design of the communication tool with parents and HCPs, (4) prototype—develop the design of Chloe’s card, and (5) test—pilot test in clinical practice (Figure 1).

### 2.3. Data Analysis

Thematic analysis of the interview data collected in phase 2 and 3 was undertaken [31]. Microsoft Word and Excel 365 software packages were used to manage the data. All data were read several times by two researchers to obtain an overall understanding and identify codes. Initial codes were independently generated by two researchers from the transcripts based on the study objectives. Data were then grouped into potential main themes and subthemes by identifying patterns, assimilating codes that overlapped, and separating out distinct codes. The coded data were collated and reorganised through team discussions, allowing further refinement and review. Recruitment continued until data saturation was achieved. Data saturation was apparent when researchers noted that no new codes were identified through the analysis of the latter transcripts [32].

Survey outputs from phase 5 were reported using mean and standard deviation from Likert scales (negative 1–5 positive). Descriptive and non-parametric statistics were used to describe the data, with a *p* value of <0.05 considered significant. Analysis was carried out using SPSS v25 (IBM, Armonk, NY, USA).

## 3. Design Thinking

### 3.1. Phase 1: Literature Review

A literature review was completed to examine the evidence supporting communication between parents and HCPs during a hospital admission. A literature search protocol was developed, and studies were identified by searching the NICE Healthcare Databases Advanced Search website (https://hdas.nice.org.uk/ (accessed on 20 June 2023)); a tool for searches within multiple databases including the PsycInfo Cumulative Index to Nursing and Allied Health Literature (CINAHL) and Medline. PubMed, the Cochrane Library, and NHS Evidence were also searched, with searches adapted for each database. Forward and backward citation searching was completed. Key search terms were adapted for each database but included derivatives of patients and parents; children with medical complexity; communication and experience of care; and palliative care with searches completed until July 2022. Studies were considered if they examined being listened to/heard or focused on barriers and facilitators of communication and were not restricted to paediatrics or any methodological approach. Studies identified were reviewed using a tool developed for assessing research with diverse design [33].

#### Results

After screening, 51 papers were included in the review (Supplementary File S1 Figure S1; Supplementary File S2 Table S1) [1,16,17,34,35,36,37,38,39,40,41,42,43,44,45,46,47,48,49,50,51,52,53,54,55,56,57,58,59,60,61,62,63,64,65,66,67,68,69,70,71,72,73,74,75,76,77,78,79,80,81]; thirty-five studies focused on aspects relating to communication in paediatrics, fifteen studies described shared care models in adult care, and two tools were parent led. Key findings from these studies identified the following themes that may be pivotal for an empowerment and communication tool: (1) bidirectional sharing of information, which may be impacted by the need for HCPs to deliver task-based care achieving standards for evidence-based care, (2) honest and proactive communication and sharing of values of parents and allowing them to parent, (3) acknowledgement of parental efficacy and the importance of being known and heard as experts of their child, and (4) HCP communication between and with parents, with barriers to good communication associated with time constraints, lack of communication training, varying medical styles, and individual HCP experience [1,16,17,34,35,36,37,38,39,40,41,42,43,44,45,46,47,48,49,50,51,52,53,54,55,56,57,58,59,60,61,62,63,64,65,66,67,68,69,70,71,72,73,74,75,76,77,78,79,80,81].

### 3.2. Phase 2: Define—Qualitative Interviews

#### 3.2.1. Interviews between Parents and Healthcare Professionals

Using an interview guide with questions focused on the format, including design and process, we completed semi-structured interviews with twelve parents and twenty HCPs (including speech and language therapists (n = 2), dietitians (n = 2), doctors (n = 4), physiotherapists (n = 2), nurses (n = 10)) recruited from paediatric cardiology, neonatal, and paediatric intensive care units. The following themes were generated from the HCP consultation:

##### Healthcare Professionals’ Interviews on Factors Influencing Parents Experiences of Communication

HCPs recognise that having an infant with CHD or children with medical complexity is hugely stressful for parents. Healthcare professionals reported that good communication is essential, but is not limited to parents, as communication with other healthcare professions and domains within a hospital structure are also essential. There was a single overarching theme of HCPs influencing parents’ experience of communication, which included subthemes of (i) location of conversations, (ii) transition between units and wards, (iii) role of nurse in advocating for parents, and (iv) parents understanding feeding goals.

(i) Location of conversations

HCPs felt parents were affected by the location of conversations, with an example of daily ward rounds. Nurses and allied healthcare professionals, in particular, reported that ward rounds could be a source of distress for parents. They described watching parents listening in for snippets of information and how these were open to misinterpretation, and sometimes not even related to the parent’s child.

(ii) Communication to support transition between an intensive care unit and step down-ward

Nurses reported that some parents struggled with the transition from the intense bedside support they received on intensive care to an environment where there is less medical monitoring and support with feeding. A number of HCPs described that communication between the paediatric intensive care unit and wards were not always collaborative. Nurses and dietitians reported feeling dismissed by other professionals particularly around feeding plans.

(iii) Communication between healthcare professionals and parents

Nurses in particular demonstrated empathy towards parents and felt the need to advocate on behalf of parents. In the paediatric intensive care unit, nurses spend 12 h at the bedside developing a relationship with parents.

There was a disconnect between various healthcare professional teams, nurses, and parents. Nurses reported being overlooked, undervalued by other healthcare professionals, and often not listened to as a conduit and facilitator of knowledge and information sharing. ‘Doctors need to realise that nurses have a very unique relationship with parents and are able to speak on the parent’s behalf. They need to understand where things have been previously tried [HCP_102]’.

(iv) Avoidance of conflicting messages from healthcare professionals to parents

Nurses and allied HCPs expressed concern about mixed messages given to parents and the impact this may have on care, ‘Consultants often come and just take the nasogastric tube out, even when the nurses say they have tried to feed but the consultant wants to take the tube out and discharge home [HCP_118]’.

(v) Feeding related communication

Healthcare professionals perceived that they contribute to parents feeling a loss of control. ‘Parents become de-skilled in hospital due to not being able to feed their babies, not being allowed to feed when they want to, … nurses are instrumental in taking that role away from them. [HCP_102]’ Healthcare professionals, describe the disconnect between parental expectation around nutrition and feeding. A communication tool could be used to ‘empower the parents to talk about what they are feeling [HCP_101]’.

#### 3.2.2. Communication—Parents

Parent semi-structured interviews were conducted with 12 parents (10 mothers, 2 fathers) from PICU and Paediatric Cardiology. Interview questions focused on (i) experiences of shared decision making and communication, with reference to feeding and nourishing their child, (ii) parental roles during an admission to hospital, and (iii) exploring the need, use, and possible format of a communication tool.

Two overarching themes were identified relating to (1) parental needs around communication. Subthemes included (i) being listened to, (ii) having a clear explanation of what is happening, (iii) involvement in decision making, (iv) good communication, and (2) the impact of poor communication, with subthemes of (i) lack of control, (ii) feeling unprepared, (iii) loss of a role as a parent, and (iv) no choices around feeding and loss of maternal status. These themes are elaborated on below:

##### Parental Experiences and Needs around Communication

(i) Being listened to

Some parents reported that they were not being listened to by HCPs: ‘When we raised concerns because we thought something wasn’t right, no-one listened to our concerns [P_105]’.

(ii) Having a clear explanation of what is happening

Parents wanted conversations, particularly around feeding. ‘Each parent needs to feel that their child is an individual and they matter. I think that along with a continuous communication…… I would have really appreciated that if the dietitian left me with a really clear plan of what the feeding plan was, that would have been really helpful for me [p_101]’.

(iii) Involvement in decision making

Although parents understand that healthcare professionals are time poor, for many, their experience of communication and involvement in decision making, particularly around feeding, was piecemeal. “We want to be involved; we don’t want conversations to happen without us [P_105]”. Parents want to be kept informed and be involved in decisions that are important to them. As a result of a lack of bidirectional knowledge sharing, parents felt their expert knowledge of their child remained unacknowledged and uninvolved in the decision making process.

Parents described how they felt when their wishes about their child’s care were not considered nor involved in the shared decision making process. “Involved? Not really, because I gave birth to her, we had a minute of skin to skin, it was timed, then she was put on resuscitation, …… and, when I was allowed to go into neonatal, she already had a tube in, I was quite surprised to know that would happen [P_103]”. Parents felt especially left out of the decision making role around feeding, “It felt like once a [feeding] plan was in place it was almost un-changeable, and I felt like a broken record a lot of the time just trying to say ‘I don’t think this is working’ [P_102]”. In contrast, when parents were able to take responsibility for feeding their child again, they felt more in control and their parental status was reinstated. “When the nasogastric tube had been taken out I felt like more of a mother, I was able to connect with her, whereas if I’m honest I felt quite detached from her when the NG tube was in [P_105]”.

(iv) Choices around feeding

There were contrasting perspectives about parents’ feelings of being supported by HCPs. One couple reported that their child was receiving all the support, and yet they, as parents, were not receiving any support. “The process was pretty overwhelming, stressful and as parents we felt vulnerable [P_102]”. Another parent reported the absence of help: “I felt there was no help, I felt very alone [P_106]”. In contrast, other parents reported their experiences more positively and felt they were able to develop a good rapport with HCPs, particularly with regards to feeding and they felt supported. “I was supported to get involved in feeding, I felt supported one hundred percent” and; “I just found the team really, really good and really helpful, they just really felt for me, and they were really honest [P_104]”.

##### Impact of Poor Communication—Parents and Healthcare Professionals

Poor communication has a negative impact on HCPs and parents with subthemes of (i) lack of control, (ii) feeling unprepared, (iii) loss of a role as a parent, with related consequences, and (iv) no choices around feeding and loss of maternal status.

(i) Lack of control

Parents described the need to adjust to their situation in hospital and the ‘unbelievable shock’ at having a child with a heart condition. They describe their shattered expectations about what life would be like once their baby was born, making them feel out of control. The consequence of parental loss of control was described by one parent who said they felt as if they were “on the side-lines” [P_101] initially but felt more in control as time went on.

Parents recognised the expertise of the HCPs and their role, but were conflicted over the need to relinquish control of their child over to HCPs. This power imbalance resulted in a dynamic shift between parents and HCPs, with parents reporting that HCPs had the last say and control over what happened to their child; “It was never a consultation, it was always, ‘we know what we need to do so we’ve got our plan, and this is what we’re doing’….. And you feel like if you don’t say yes you feel like you’re not doing what’s right for your child [and that was] awful really [P_107]”.

(ii) Feeling unprepared

For many parents, they were completely unaware there may be any feeding difficulties once their infant was born. “If someone had explained to me what was happening, I would have felt part of the process and part of the journey and it wouldn’t feel as much of a loss of the things that you wanted to do as part of being a mum [P_102]”.

A parent described when HCPs took the time to explain and answer the same questions multiple times around the child’s medical condition. “They explained everything in a way that we could understand without the big medical names. So, when they tried to talk to you they talk to you as humans rather than as patients, they talk to you as a normal person… that made it easier for us to accept everything [P_101]”.

(iii) Loss of a role as a parent

Parents felt their role had been taken away from them and re-defined, “Parent decisions are no longer yours to make” [P_105] or taken away from them “Parental role was totally taken away, it made me feel extremely frustrated and sad” [P_104]. Parents felt unheard and there was little acknowledgement of their role and expertise as a parent “…most of the medical professionals …. assume they will know more than you, and therefore the decision that has been made is better than the decision that you could make as a parent….. [P_108]”.

(iv) No choices around feeding and loss of maternal status

The experience of communication around feeding choices is where parents reported that they were not listened to or consulted, and unable to ask. Parents also described their feeling of loss when their plans to breastfeed were taken away and how distressing this was for parents. “As a mum who really wanted to breastfeed, I felt like that was totally taken away from me and I’m so bitter about it even two years on, I feel like crying…… [P_103]”.

This resulted in parents feeling unheard and disempowered, with their viewpoint around feeding often disregarded. “Basically, I was told what would happen….I said I’d like to breastfeed and then they said ‘well she’ll get really tired really quickly so in order for her to get the calories and the feed that she needs, we will feed her……”. In contrast, other parents had a positive experience and felt involved in the decision making and respected their viewpoint on their child; “….it was really nice to know that they appreciate that as a parent you also know your child [P_104]”.

### 3.3. Phase 3: Ideate and Iterate

From the qualitative interviews, parents and HCPs alike felt a communication tool would be useful and identified the need for a small business card that could be discretely given to HCPs at the bedside. Based on the qualitative feedback from phase 2, we co-designed and co-created a communication tool with parents, healthcare professionals, and parent charities. The name for the communication tool, Chloe’s card, arose from a bereaved mother’s lived experiences and close cooperation in the study. Initial designs of Chloe’s card were shown to parents (n = 5) and HCPs (n = 5) who did not participate in the qualitative interviews. During these interviews, parents and HCPs were also asked what format a communication tool (Chloe’s card) may take, the information it should contain, and what processes should be considered. They were also asked whether the proposed name of the card would be acceptable. Iterative changes were made to the design (content and format) and process of the tool after each interview until thematic saturation was achieved.

### 3.4. Phase 4: Develop the Prototype

Five iterative design changes were made to the overall design of Chloe’s card, supported by a professional communication agency (Raised by Wolves https://rbw.london/ (accessed on 6 January to 30 July 2022)). Key perspectives underpinning the tool included: (1) parental needs around communication, (2) being involved in decision making and care, and (3) choices around feeding and the importance of the parental role (Figure 1).

#### 3.4.1. Parents—Card

Parents felt the initial wording of ‘I’m not being heard’ or ‘I am not being listened to’ would be difficult for them to use, as they would not want to appear rude. Parents reported that a series of questions for HCPs would be useful to prompt conversations around care. HCPs agreed with these suggestions and positively viewed the idea of an “Ask me card” as a way of finding out more about parents and their children. The final design included a space for parents “to tell us what you know and ask us what you don’t” with space for parents “to list what you would like to discuss here”, with a date and time for these concerns to be reviewed. The center of the card contained emoticons that parents and HCPs reported were important to them, including concerns about (i) pain/symptoms, (ii) feeding/eating, (iii) weight/growth, (iv) medicines, (v) options/decisions, (vi) how you can help?, (v) family siblings, and (vi) worries and coping. The reverse side of the card sought to reassure parents that ‘It’s ok to say…. If there is something worrying you or you don’t understand. Please tell us’.

#### 3.4.2. HCP—Card

**Figure 2:**
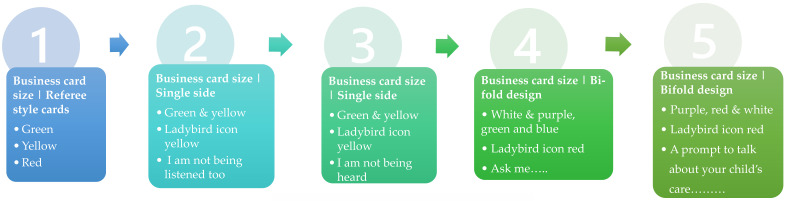
Iterative designs of Chloe’s card.

The card contained the following three prompt questions for HCPs: (1) What should we ask you about? (2) What have we NOT asked about? (3) Anything else? Have we talked about everything you hoped we would? The HCP card also included information about what the tool is for and information about Chloe’s card, including why is it useful (i.e., to start a conversation, to build relationships, pausing to hear what is important, listen to worries and uncertainty, explore options and decisions) and how to use Chloe’s card (i.e., give Chloe’s card to all parents on admission, check in with parents about Chloe’s card within 24 h of admission, action anything that require escalation, and revisit with parents each week). HCPs reported they would value a quick reference card to remind them of useful prompt questions as well as the process for escalation. The HCP tool was developed in contrasting colours of light purple with mauve writing (Figure 2). All parents and HCPs found the name “Chloe’s card” acceptable and related to the human aspect associated with its development.

### 3.5. Phase 5: Pilot Testing of Chloe’s Card

#### 3.5.1. Testing the Intervention—Surveys

For the final cycle (phase 5), we completed a four-week pilot study in three clinical areas, within the hospital, to test our assumptions based on using the shared decision making tool in clinical practice.

#### 3.5.2. Outcome Measures

A validated parental communication survey [82] was used as an outcome measure to assess the impact of the intervention, containing questions on (i) the sharing of information, (ii) relationships with doctors and allied health professionals, (iii) making decisions, (iv) dealing with uncertainty, (iv) taking care of your child, (v) attention to your emotions, (vi) providing validation, and (vii) supporting hope.

In addition to this, a short survey was developed and piloted among three HCPs and two parents to gain a better understanding of what parents and HCPs thought of Chloe’s card. The survey contained questions on (i) using the card as part of clinical practice/admission (dichotomous yes/no) and three open-ended questions, (ii) what worked well, (iii) what did not work well, and (iv) what should be changed.

#### 3.5.3. Preparation for Testing

##### Staff training

Two months before the launch of Chloe’s card, staff training was provided by the research team on how to use the card. Training took place during scheduled staff meetings, in addition to nursing handovers, as well as a series of emails sent to “all” staff within each of the clinical areas to ensure information was widely disseminated. A short video detailing the tool was also included on the Staff Intranet, on a dedicated Chloe’s card page. The training included instruction on how to use the card as part of the HCP quick reference card.

##### Promotion of the intervention

Prior to the launch of Chloe’s card, posters were placed in clinical areas as well as dwell areas for staff and parents.

##### During the testing period

All parents were given Chloe’s card on admission as a prompt to talk about their child’s care. At the beginning of each week, researchers followed up with HCPs to trouble shoot problems as well as to talk to parents about the use of the card. HCPs and parents were encouraged to complete an anonymous feedback survey on their thoughts of Chloe’s card in practice. In addition, parents were asked to complete an anonymous survey, which was available online.

##### Debrief following the testing period

Debrief sessions were held with staff groups in each of the clinical areas, comprising of consultant meetings, senior sister meetings and other staffing groups, as well as parents for them to provide qualitative feedback on what went well and what aspects of the tool need to be changed.

### 3.6. Results of the Testing Phase

During the 4-week testing period Chloe’s card was given to 135 families within Neonates, PICU, and Paediatric Cardiology.

#### 3.6.1. Feedback on Chloe’s Card

**Figure 3:**
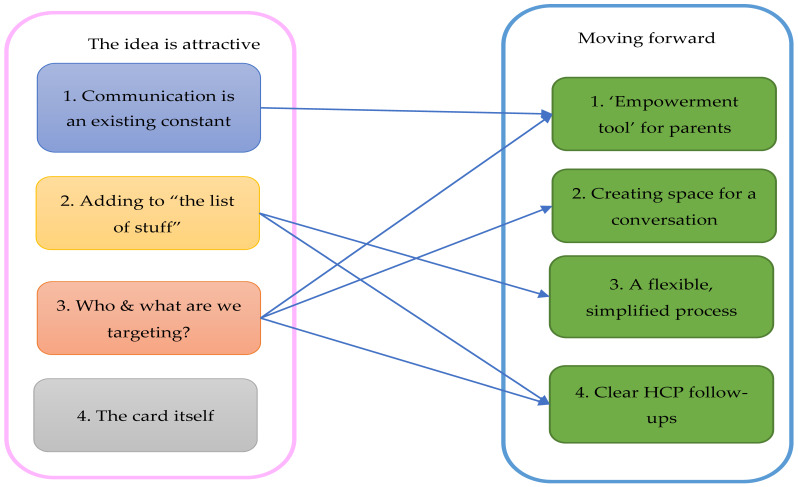
Theme map linking feedback themes ‘the idea is attractive’ with ‘moving forward’ themes.

Following the 4-week pilot study, a thematic analysis was completed on the feedback from the pilot study of Chloe’s card received from HCPs and parents, both verbally and via qualitative text responses from the survey. Feedback on the tool was given in a variety of contexts, including individual conversations with parents/caregivers, researcher meetings with consultants, and small feedback groups with HCPs (n = 121) from various backgrounds. There were four overarching themes relating to the use of Chloe’s card in clinical practice: (1) communication is an existing constant, (2) adding to “the list of stuff”, (3) who and what are we targeting, and (4) the card itself (positive and negative) (Figure 3). Chloe’s card was perceived as a good idea….“In principle it is a great idea—most of our complaints arise from parents not being heard, so the necessity is obvious” [HCP_PICU_201], and prior to the pilot was keenly awaited by the HCPs in the clinical areas. However, aspects of the implementation of the card in practice were flawed and some HCPs found the concept of a communication tool offensive, as communicating well was something they took pride in. Both contextual, i.e., who is the card for and how do HCPs perceive it, will support better communication and process-related factors, i.e., when parents should be given the card, and who and when checks in are included, which may prevent successful implementation…. “The worry for us is that it could be just another clever idea… staff are not quite sure what to do with it” [HCP_Neonates_205] (Figure 3).

##### The Idea Is Attractive

From the interviews, we found that parents and HCPs alike reported the need for a tool such as Chloe’s card, and that it would be an opportunity to explore the use of shared decision making further. One statement that stands out from the parent interviews is ‘The card isn’t empowering, what’s empowering is that somebody actually listens and actually acts on what you’re asking’ [P_105], and from HCPs: ‘Why don’t we involve patients in decision making? [HCP_112]’.

In general, the feedback from participants was viewed initially as an acceptable tool and idea that was keenly adopted by the healthcare provider.

“In principle it is a great idea—most of our complaints arise from parents not being heard, so the necessity is obvious” [PICU_Consultant meeting].

“It is a brilliant idea… problem with the current pilot is that no one understands what to do” [Neonatal_Senior Sisters meeting].

##### Perceptions of Good Communication

Communication is constant on the wards and being good at communication is part of HCP identity. However, the need for communication training and tools varies by role level, with more senior staff identifying that Chloe’s card is not useful for them but may be for other junior staff lacking in experience and confidence in communicating with parents.

“We communicate really well with parents and so we don’t feel the card is necessary” [Neonatal_Senior Sisters meeting].

“It may be useful for junior members of staff as experienced staff know what to do with regards to communication”.… “It is sad we have to have this tool” [Neonatal_Senior Sisters meeting].

##### Adding to the ‘List of Stuff’ to Do?

In general, parents reported that their experiences of communication with HCPs were positive. They talked about this both generally and in relation to Chloe’s card. Some parents reported not needing the cars, whilst others found it helpful. Adding to administrative burden was a clear issue to HCPs in a time-constrained and stressful work environment. This was also highlighted as an issue for busy and stressed parents who have a lot of information to take in and a lot of forms to fill.

“People are just about holding on—it is just another thing to do” [Neonatal_Senior Sisters meeting].

Within the ‘list of stuff’, HCPs also expressed worries that parents would use Chloe’s card similarly to an existing escalation process/tool.

“Are we not opening up a can of worms? For parents to find fault and complain about us?” [PICU_Nurses meeting].

Firstly, the issue of who to target was important. There is a clear distinction between parents who are high versus low in communication confidence/competence. For parents high in confidence and competence, this card was not seen as necessary. More quotes are provided in ‘moving forward theme 1′ on how the card could be adapted to target different categories of parents.

“She is very extroverted so feels that she always asks everything, but she can understand that for people who are more shy this could be a good tool” [Parent, Neonates, via feedback].

##### Who and What Are We Targeting?

HCPs highlighted that the scope (or ‘what’) was too broad. There is a ‘communication continuum’, and it was unclear whether Chloe’s card was task, process, or patient focused. The matrons also highlighted the nature of news changing over time—the ‘bad news’ continuum. The broad application of the card becomes confusing in the context of very busy/burnout staff. Thirdly, the issue of who is implementing the cards (and follow-up) was raised.

There was also some speculation about exactly where/in which department Chloe’s card should be used. Comments highlighted settings in which the card may be more appropriate, and where follow-up could be conducted more easily.

“It may be more useful elsewhere and it could be PICU ensure parents have a card and whether there is anything that needs to be picked up from parents. It may be more useful to be given to parents in orther environments, particularly in as part of step down—where parents are weakly monitored compared to the high intensity of PICU” [PICU_Consultant meeting].

“Champions are necessary, then it will happen by osmosis” [Neonatal_Senior Sisters meeting].

##### Appearance and Content of the Card

Feedback on the cards appearance and content was overall positive. People felt the card was attractive. Purple is a good colour to move away from the hospital/NHS blue [Parent_Cardiac]. The prompts/pictures are very useful [Parent_Neonates]. Posters were written in a way that is very feminine, when babies are not gender-defined yet [Neonatal_Senior Sisters meeting].

One nurse commented, “Calling it ‘Chloe’s card’ automatically makes parents aware of a death and a situation of lack of parental communication. In my opinion this heightens anxiety and disgruntlement. It should be called a generic parental communication card..”. [Nurse_Neonates].

#### 3.6.2. Moving Forward

Participants identified that the card would be potentially useful to target parents who are less confident or who struggle with communication due to language or communication barriers, or who lack confidence/are shyer/are less extroverted.

##### Empowerment Tool for Parents

Chloe’s card as an empowerment tool has the dual benefit of changing the way HCPs view the tool. Reducing the emphasis on communication skills and moving toward a parent-focused initiative may improve the perceived appropriateness of the tool by HCPs. Survey comments (‘what do you think?’) indicated that the prompts were helpful for parents to remember which questions they wanted to ask:

“Non-native English speaker mum on E1 [cardiology ward] felt better about speaking to staff rather than writing down questions in card because she is not comfortable writing English” [Parent_ Cardiac].

##### Creating Space for a Conversation

HCPs and parents felt there was a clear patient focus (rather than a process or task focus), i.e., this is not about pushing forward with treatment-related tasks; there are processes that exist for this. Many of the quotes talked about a ‘reset’ or ‘pause’ to establish a line of communication. The card was viewed as a ‘good conversation starter,’ creating space for an in-person conversation following stressful scenarios, focusing on the ‘tell and ask’ slogan.

“Attractive idea… If it facilitates a communication reset, it is helpful” [Cardiology_Consultants Meeting].

##### A flexible Simplified Process

Addressing the concern that Chloe’s card adds to ‘the list of stuff’ HCPs have to do called for a simpler (timesaving) process needed for the cards to be successful. Ad-hoc vs routine use also need to be considered moving forward. Feedback indicated that some flexibility is needed, as another routine process only worsens staff burden.

“It may be a good idea to have bedside boards with the same idea as the card to prompt questions” [Parent_Cardiac].

“Would only do it if I had the time, not as a routine procedure” [Neonatal nurse].

##### Clear HCP Follow up

There were conflicting ideas about who should be addressing issues raised on the card and who should be following these up. This links to ‘the list of stuff’ and ‘who and what’ themes, where clear personnel and processes are needed to ensure the card does not become lost in staff workload. Junior nurses (and other junior staff) were enthusiastic, and feedback indicates that they could deliver Chloe’s card successfully if supported by senior staff to help answer parent’s questions thoroughly. There was a lack of confidence from other HCPs in junior nurses’ communication ability, but they felt that Chloe’s card had the potential to help with this, as it could be implemented as part of junior staff training (Figure 3).

#### 3.6.3. Communication with Health Care Professionals

Twenty-nine parents completed the communication survey, and although there was generally a high level of satisfaction ceiling effect within the survey with a mean score of 4.06 ± 0.15 out of 5, there were several questions reporting lower scores. These questions were in the following domains: (i) sharing of information, (ii) relationships with doctors and other HCPs, (iii) dealing with uncertainty, (iv) taking care of your child, (v) attention to your emotions, (vi) providing validation, and (vii) supporting hope. With regards to communication, parents found that information was easiest to obtain from nurses 4.8 ± 0.4 compared to doctors 4.0 ± 0.9, *p* < 0.05, dietitians 4.1 ± 0.8, *p* < 0.05, and other HCPs 3.91 ± 0.8, *p* < 0.05, suggesting a tool between nurses and parents could be of further support (Supplementary File S3 Table S2).

#### 3.6.4. Parents and HCPs Views of Chloe’s Card

Twenty-seven parents and twenty-eight HCPs completed the short survey, which sought to understand their views of Chloe’s card. The majority of parents 81% (n = 22/27) said it was easy to use, with 78% (n = 21/27) parents finding it useful in helping start a conversation and 85% (n = 23/27) felt the tool required little explanation to be able to use it. In contrast 79% (n = 22/28) of HCPs said it was easy to use, 61% (n = 17/28) found it helpful in starting a conversation, 57% (n = 16/28) felt it reduced parental worries, and 57% (n = 16/27) felt the tool could support building relationships with parents.

## 4. Discussion

Using design thinking, it was possible to develop a prototype of a tool to assist shared decision making and communication between healthcare professionals and parents within three busy clinical environments and children with medical complexity. Healthcare professionals and parent participants in this study reported that Chloe’s card acted as a ‘good conversation starter’, creating space for an in-person conversation following stressful scenarios, supporting bidirectional sharing of knowledge. There are noteworthy interventions that attempted to deliver a similar approach through improved communication by emphasising the parent voice. Examples include semi-structured reflection sheets to help prepare parents for dialogue with HCPs [83], and ‘Listening to you’, which sought to develop communication bundles and resources to support communication between parents and HCPs [37]. Chloe’s card adopted a design thinking approach to the development of the communication tool, the principle of which involves consideration of key challenges (emphasising and defining), with suggested solutions (ideation), proceeded by a process of testing, evaluation, and iteration [84]. Lomborg et al. [85] describe the use of a design thinking approach to develop seven conversation cards as part of an intervention for individuals with type 2 diabetes. Nurses who completed annual diabetes status visits reported the use of the cards placed a greater emphasis on shared agenda setting, moving the focus away from a checklist to meet data collection requirements. The adoption of design thinking in this study permitted the development of various designs subjected to user testing. The preferred design was complete with bold graphic design favoured by stakeholders and is similar to the way in which Chloe’s card was developed. Tools where a design thinking approach was used may be more engaging, and were suggested to change the focus of HCP contacts with patients from a data-focused task to one that is more personalised to the healthcare users, promoting patient-centered care [85]. However, although shared care and communication tools may improve health outcomes, reported studies were unable to address the challenges of defining and identifying appropriate outcome measures to determine efficacy of the tool’s effect [37,85], limiting our understanding of how these tools may work to improve patient outcomes. An example is the widespread use and adoption of “hospital passports” containing pertinent information relating to the health, communication, and support needs of individuals with intellectual/learning disabilities [86]. Hospitals are used by parents and children with additional needs to talk to healthcare professionals [87]. Hospital passports were shown to improve communication supporting better outcomes, by supporting service user autonomy, reinforcing healthcare, service user partnerships, and supporting person-centered care [88]. Despite these and other efforts to improve communication, evidence to support efficacy and overall acceptance is sparse. For example, a review of hospital passports in the UK found more than 60 different types were in use, with considerable variation in format, length, and type of information collected [86,88]. These variations may impact the effectiveness of the tools, especially with regards to defining the purpose of the tool, content, who owns it and how it is used, the role of family caregivers (including parents), and challenges for healthcare professionals in accessing important information. The authors of these studies suggest that in order to ensure that patient/parent-held communication tools are useful, further research and engagement with stakeholders is required [86,88]. A parent’s ability to access communication tools is not limited to cognition, but also includes other factors, such as vision, hearing, reading, writing, and language [88,89,90]; for example parents with a learning disability may experience greater difficulties with problem, solving, verbal expression, comprehension, and social communication [91] with regards to the care of their child. As such, parent-held communication tools may help to support parents with a learning difficulty or additional communication needs to articulate concerns through the use of alternative text or language, imagery, or spoken word relating to the care of their child [90]. However, more research is required with regards to the (i) design and format of tools (i.e., paper or digital), (ii) language (i.e., literacy and non-English speakers), (iii) differences in communication styles gender (i.e., fathers and months), (iv) as well as how these tools could be standardised and incorporated into a trusted digital health environment (i.e., electronic patient records). Findings from these studies mirror ours, as during this development phase of Chloe’s card, we identified a number of important outcome measures (parents and staff) to report the potential efficacy of using Chloe’s card on communication, empowerment, and staff confidence, which will be taken forward into a larger prospective study.

The use of Chloe’s card also elicited mixed responses, in particular, from senior nursing staff, who perceived that the tool undervalued their skill and expertise in communication with families. Similar to our findings, Heath et al. [37] report mixed HCP views, with some staff reporting the tools being of great value, whereas others perceived the tools to be “undermining and offensive”. The authors postulated that HCPs felt they were already competent and confident communicators who were able to escalate parental concerns. Feedback on the use of Chloe’s card similarly suggest tools developed to support communication may fail to acknowledge the communication skills of experienced HCPs, whilst failing to address training needs of less-experienced HPCs. A systematic and meta-analysis of tools supporting communication identifies a lack of standardised processes and training, leading to poor parental experience of care [20] and HCPs feeling burdened by additional tasks associated with these tools [7,19]. However, whatever tool is developed, parents must feel able to first voice their opinions and that their concerns will be heard and actioned, and in circumstances where reflexivity may not be embedded within staff training, listening opportunities may be missed. Parent (person)-centered communication is where parents are recognised as unique with their children having individual care needs and viewed as collaborators in the care process [38]. Specific elements of person-centered care that were associated with positive outcomes are (i) patient-orientated interventions—patient-centered approach and self-management, (ii) professional intervention—training healthcare providers including teaching and simulation, (iii) organisational interventions—enhancing an interdisciplinary approach, supporting decision processes and an evidence-based approach, providing case/care management, and integrating information technology [92]. Chloe’s card sought to support patient-centered care, although the result from our testing phase suggests that further education and training around these concepts require development.

### 4.1. Impliactions for Practice and Future Research

This design thinking project aimed to co-create and co-develop a communication tool for parents with CHD and complex illness. The initial design of Chloe’s card took the form of a small business card for parents to use during an inpatient admission, with a complimentary version for HCPs to serve as a quick reference guide on how to use the tool in day-to-day practice. During this project, parents expressed value around being empowered and supported the idea of having a tool to help them achieve this. HCPs saw value in a tool that may support shared care and bidirectional communication, and that Chloe’s card was a useful prompt for care. However, there were several areas for improvement as suggested by participants, relating to the inclusivity and accessibility of Chloe’s card for all parents and that further work will be required to ensure it is available in different formats and languages, ensuring accessibility to all parents within all health and social care settings. For this study, we had a gender imbalance with limited representation of a fathers perspective. However, as part of a larger study, understanding communication from the perspective of fathers and mothers will be important. This point is highlighted by Jones et al., who considered effective nurse communication in a neonatal intensive care unit. The authors reported that for mothers, good communication involved personalised interactions and the consideration of the role mothers and the nurses have in caring for the infant. In contrast, for fathers, good communication occurred when information was concise, accurate, and consistent.

HCPs reported that, Chloe’s card alone as a prompt for shared care and better communication was insufficient to drive the changes required to improve communication between parents and HCPs. Some HCPs felt Chole’s card undermined some HCPs expertise, but may be of support to junior colleagues. Disenfranchisement is common in parents and children with life-limiting conditions, who describe feeling disempowered, vulnerable, and excluded from treatment decisions and unable to provide parental care for their child [55]. Parents also stressed the need for self-efficacy, honesty, and importantly, to be recognised as the expert of their child [56]. Parents of children with life-limiting conditions also value the idea of both collaboration between themselves and the need for specialist care from health care professionals along with simultaneous empowerment [56]. As such, more needs to be accomplished to hear and recognise the role of the parent, with particular reference to feeding and empowering parents to assert their role as being the expert of their child, as reported by parents.

Several studies identify the need for increased training in communication skills for healthcare professionals when implementing models of shared care [92,93], and that nurses often lacked confidence in communication with parents [83], or were overconfident in their perceived communication skills around teaching parents compared to parents’ perception of their skills [94]. However, there is very little communication skills training for healthcare professionals, other than those associated with delivering bad news [95], and even fewer opportunities to learn these skills as part of a team learning together within a clinical setting. Simulation-based learning experiences can be used as part of team building to supplement clinical experiences supporting healthcare professionals to build competencies in person-centered communication [63,96]. To build on this foundational work, we plan to develop an online training program to incorporate reflective practice as part of Chloe’s card, which will seek to provide a transformative approach to parent (person)-centered care [38,63]. In addition, we plan to develop a clinical simulation training program using role-play scenarios and educational packages for units to train together as part of a wider implementation toolkit for Chloe’s card to support HCP communication skills around shared care decisions with parents in relation to goals of care including those aspects important to parents [63].

### 4.2. Limitations

Our findings from this small pilot demonstrate that whilst many parents and HCPs value the idea of Chloe’s card, HCPs did not always view the benefit of using it in clinical areas, and although parents positively viewed the idea of Chloe’s card as a prompt to deliver shared care, some felt unsure how to use it. This project, using a design thinking approach, was completed in a small number of clinical areas that have high levels of HCP input with 1:1 and 1:2 nursing, so it may not represent the viewpoints of parents and HCPs in less well-resourced environments. Further work is required to (i) develop Chloe’s card, including online training for how to use the tool from the perspective of parents and HCPs, (ii) development of a digital version of the communication tool, including alternative language options for parents where English may not be their first language, (iii) identification of outcome measures to test the efficacy of the tool to improve parent and HCP-reported outcomes, and (iv) consider how gender may impact the parental need with regards to a communication tool. This and other related work in the field of parent/person-centered care is an important step towards supporting parent/person agency with regards to developing bidirectional sharing of information, promoting pausing, listening, and hearing amongst HCPs about what is important to parents and their children in terms of their healthcare.

## 5. Conclusions

During this project we were able to develop a communication tool, Chloe’s card, with the purpose of acting as a prompt for a child’s care during an inpatient admission. The concept of Chloe’s card was acceptable to parents and HCPs. However, as a standalone tool, Chloe’s card is unlikely to be sufficient to improve communication between parents and HCPs. Further work is planned, as part of a larger study, to further refine this communication tool as well as to develop clinical simulation training for HCPs and information for parents. As part of a future study, factors that will need to be addressed will include accessibility, including design and format of tools, language and literary, differences in communication styles gender, as well as how these tools could be standardised and incorporated into a trusted digital health environment (i.e., electronic patient records).

## 6. Patents

Chloe’s card is a registered trademark copyright 2022 INO95 (Ref UK00003878521).

## Data Availability

Data is unavailable due to privacy restrictions.

## Supplementary Materials

The following supporting information can be downloaded at: https://www.mdpi.com/article/10.3390/healthcare11202706/s1, Supplementary File S1 Figure S1: Prisma Literature flow diagram, Supplementary File S2 Table S1: Characteristics of studies included in the literature review, Supplementary File S3 Table S2: Communication Survey results. Supplementary File S4: Chloe’s card—What do you think about the tool?
