# Supplementary material for: The Development of a Communication Tool to Aid Parent-Centered Communication between Parents and Healthcare Professionals: A Quality Improvement Project
Source: Healthcare (Basel). 2023 Oct 10;11(20):2706. doi: 10.3390/healthcare11202706 (PMC10606263; doi:10.3390/healthcare11202706)
Supplement: Supplementary file 1 [file healthcare-11-02706-s001.zip › healthcare-2649078-supplementary.pdf]

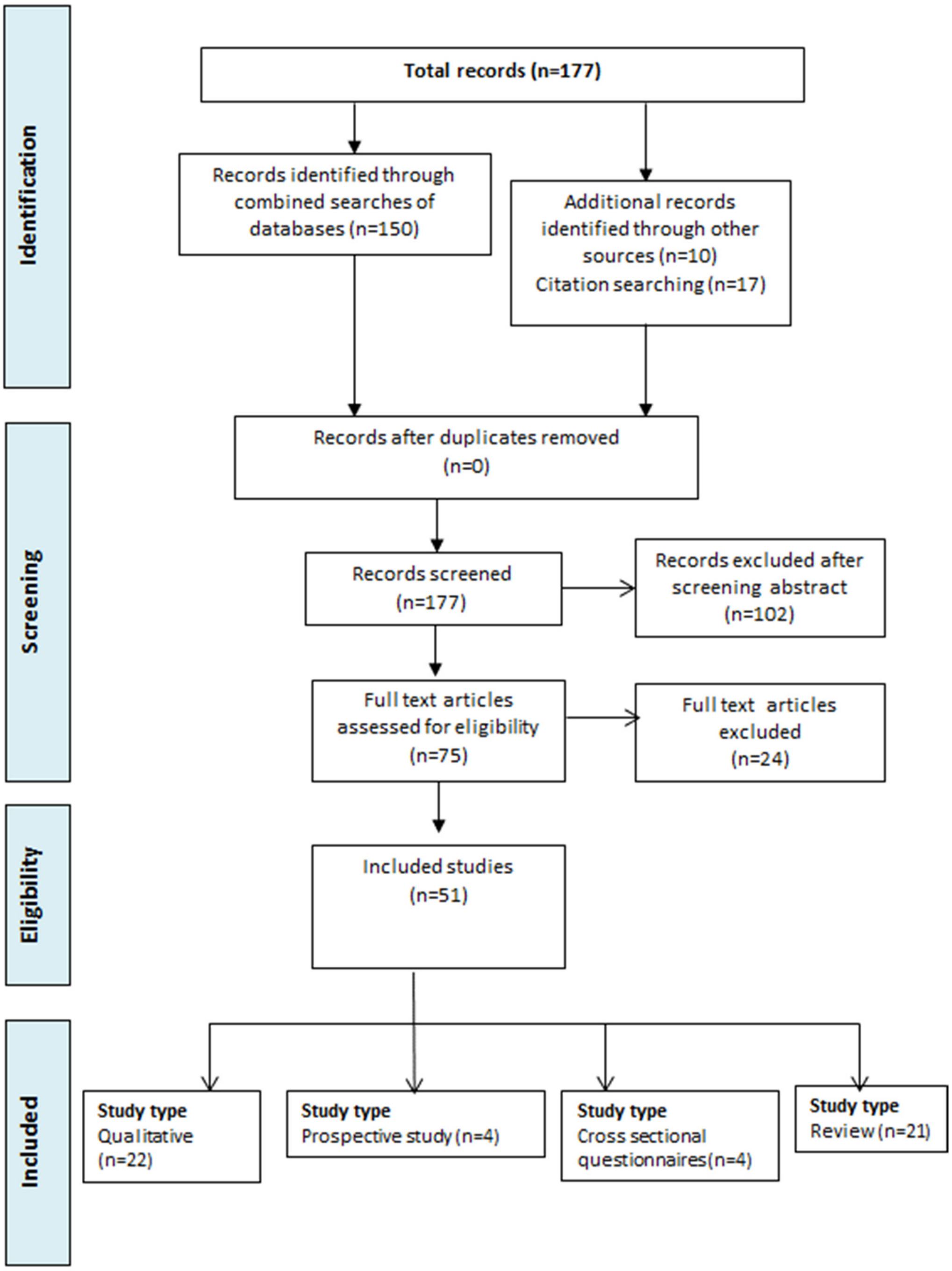

Supplementary File S1 Figure S1. Studies included in literature review.

Supplementary File S2 Table S2. Articles reviewed as part of the literature review.								
**First author & date**	**Journal**	**Aim**	**Study Design**	**No. of interviews/ Surveys/ observations**	**Adult/ Paediatrics**	**Study population**	**Theme**	**Findings**
Janvier (2014) (1)	Seminars in Perinatology, 38;(1):38-4638;(1):38-46	To describe the use of mnemonic SCOPIE	Review / Observation	N/A	Paediatrics	Doctor/ parent interaction	End of life	SCOPIE (1) What is the Situation? Is the baby imminently dying? Should withholding or withdrawing life-sustaining interventions be considered? (2) Opinions and options: personal biases of HCPs and alternatives for patients. (3) Basic human interactions. (4) Parents: their story, their concerns, their needs, and their goals. (5) Information: meeting parental informational needs and providing balanced information. (6) Emotions: relational aspects of decision making which include the following: emotions, social supports, coping with uncertainty, adaptation, and resilience.
Denis-Larocque G (2017) (2)	Intensive & Critical Care Nursing, 43;149-155	To explore nurses' perceptions of caring for parents of children with medical complexity in the Paediatric Intensive Care Unite (PICU).	Qualitative study design/ Interviews	10	Paediatrics	Nurses	Children with medical complexity (CMC)	Themes of PICU nurses' perceptions: 1) "Thrown to the wolves": Adjusting to a new caregiving role; 2) "Getting to know each other": Merging caregiving roles; 3) "Keeping connected": Working to preserve the partnership
Quigley L (2014) (3)	BMC Health Services Research, 14;283	To identify the barriers and facilitators of information sharing for CMC across providers, care settings, and families.	Qualitative study design/ Interviews	43	Paediatrics	Nurses	CMC	Barriers to information sharing were related to one of three major themes; 1) the lack of an integrated, accessible, secure platform on which summative health care information is stored, 2) fragmentation of the current health system, and 3) the lack of consistent policies, standards, and organizational priorities across organizations for information sharing. Facilitators of information sharing were related to improving accessibility to a common document, expanding the use of technology, and improving upon a structured communication plan.
Barbarin OA (1984) (4)	Journal of Community Health, 9;(4): 302-13	To explore with parents of children with cancer the behaviour of medical staff members with whom they interacted.	Qualitative study design/ Questionnaires	74	Paediatrics	Children	CMC	Seven distinct dimensions of staff behaviour relevant to their relationships with parents were identified; 1) information transmission, 2) clarity and honesty of communication, 3) acceptance of parental efficacy, 4) resolution of conflicts, 5) personal contact with parents, 6) empathy with the child, and 7) staff competence.
Orkin J (2020) (5)	Pediatrics, 145; 3	To develop an in-depth understanding of the advance care planning experiences from the perspectives of both parents and healthcare professionals (HCPs) of children with medical complexity	Qualitative study design// Interviews	25	Adult	HCPs/Patient	End of life	Interviews revealed 4 major themes and subthemes (brackets): (1) holistic mind-set, (2) discussion content (beliefs and values, hopes and goals, and quality of life), (3) communication enhancers (partnerships in shared decision-making, supportive setting, early and ongoing conversations, consistent language and practice, family readiness, provider expertise in advance care planning (ACP) discussions, and provider comfort in ACP discussions), and (4) the ACP definition.

Radu M (2022) (6)	Journal of Community Genetics, 13;(3):293-302	To explore carers' and young people's needs when living with long term conditions, as well as the views of the HCPs supporting them.	Qualitative study design/ Interviews	30	Paediatric	HCPs/Parents	CMC	Four main themes emerged: (1) Acceptance takes time refers to the often long and challenging process of adapting to the diagnosis and living with the condition; (2) Close guidance captures the importance of specialised and long term guidance in accessing and managing the complexities of the medical system; (3) Open communication shows the families' need to be collaboratively connected to healthcare providers and other families facing similar difficulties; and (4) Long-term support underlines the importance of long term formal and informal support strategies.
Morse BL (2021) (7)	Pain management Nursing, 1(2):169-176	To understand parent perspectives to improve pain practices.	Qualitative study design/ Interviews	25	Parents	Parents	Pain	Major themes included: 1) pain experiences, 2) confidence in caregivers, 3) parents are partners, 4) proactive communication, and 5) a spontaneous theme, "they can hear us." Sub-themes of pain experiences: emotional pain and challenges identifying.
Verberne LM (2021) (8)	European journal of Pediatrics, 180;(3):949-957	To explore how parents and HCPs anticipate the future of the child and family in paediatric palliative care.	Qualitative study design/ Interviews	101	Paediatric	HCPs/ Parents/ Children	End of life	Three themes: 1) goal-directed conversations, 2) anticipated care, and 3) guidance on the job.
Jones AH (2020) (9)	Hospital Pediatrics, 10;(4):325-330	To evaluate communication patterns of various clinician types, including the length of communication encounters and the number of provider interruptions at the time of admission.	Prospective observational study/ Observation	45	Paediatric	HCPs/Parents	Care	Qualitatively, residents had more challenges coping with distractions, acknowledging parental emotions, and aligning with parental goals compared with other clinician types. Doctors (trainees) spend significantly more time with families and 1) burdened with frequent interruptions and 2) may require additional communication skills to manage relaying complex information while simultaneously responding to parental emotions.
Birchley G (2022) (10)	BMJ Paediatrics Open, 6;1	To develop a consensus approach to the management of complexity among HCPs, using a modified Delphi process.	Modified Delphi consensus/ Questionnaires	99	Paediatric	HCP	CMC	Seven thematic areas: 1) standardised approaches to communicating with families; 2) processes for interprofessional communication; 3) processes for shared decision-making in the child's best interests; 4) role of the multidisciplinary team; 5) managing professional-parental disagreement and conflict; 6) the role of clinical psychologists; and 7) staff support.
Nicholas DB (2016) (11)	Journal of Social work in end-of-life & palliative care, 12;(1):126-44	To understand the experiences of fathers with a child diagnosed with a life-limiting illness (LLI).	Qualitative study design/ Interviews	18	Paediatric	Parents	End of life	The overarching themes were 1) stresses, 2) means of coping, and 3) perceived needs for support.
Gill PJ (2022) (12)	JAMA network open, 5;(4):e229085	To prioritize unanswered research questions in paediatric hospital medicine from the perspectives of young people, parents/caregivers, and health care professionals.	Modified Delphi consensus/ Questionnaires	188	Paediatric	HCPs/Parents/ Caregivers/ Children	Hospital	Questions focused on care of special inpatient populations (CMC); 1) communication, 2) shared decision-making, 3) support strategies in the hospital, 4) mental health supports, 5) shortening length of stay, and 6) supporting Indigenous patients, 7) parents/caregivers, and families.

Mackay L (2020) (13)	Journal of Pediatric Nursing, 53;14-21	To understand contemporary experiences of paediatric healthcare professionals' (HCPs) caring for hospitalized Medically Fragile Infants (MFI) and their parents.	Qualitative study design/ Interviews	26	Paediatric	HCP	CMC	HCPs encountered barriers to establishing relationships with parents, including: 1) intricate nature of MFI, 2) lack of social supports, 3) inconsistency, 4) moral distress, 5) burnout, and 6) struggle to gain control. HCPs utilized strategies to establish relationships with parents, including: 1) normalizing and building parental confidence, 2) tailoring care and being flexible, 3) providing parent care, and 4) optimizing communication.
Barnard C (2019) (14)	Hospital Pediatrics, 9;(1):39-45	To highlight factors that may contribute to communication crises through the characterization of these circumstances.	Qualitative study design/ Interviews	37	Paediatric	HCP	Hospital	Three themes and 11 subthemes (communication crisis risk factors) were identified: (1) health care team factors (communication skills, care processes, and interprofessional communication), (2) family and/or parent factors (language or cultural barriers, mental health conditions, socioeconomic factors, and beliefs), (3) patient factors (acute condition, unclear diagnosis, unstable condition, and medical complexity). A core theory emerged: parent trust in their HCP significantly impacts the therapeutic relationship and can mitigate communication crises despite the presence of risk factors.
Wigert H (2013) (15)	BMC Pediatrics,13;71	To describe strengths and weaknesses of parent-nurse and parent-doctor communication in order to improve our understanding of parents' communication needs.	Questionnaires	270	Paediatrics	Parents	CMC	Training doctors and nurses in communication skills, especially, 1) how to meet parents' emotional needs better and 2) creation of a framework for the parents of what to expect from neonatal intensive care unit (NICU) communication might also be helpful.
Rennick JE (2019) (16)	BMC Pediatrics,19;(1):272	To explore the experiences of parents of CMC during PICU admission.	Qualitative study design/ Interviews	17	Paediatrics	Parents	CMC	Four themes were identified: (1) "We know our child best;" (2) When expertise collides; (3) Negotiating caregiving boundaries; and (4) The importance of being known.
Derrington SF (2018) (17)	Pediatrics, 142;S187-S192	To explore shared decision-making (SDM) between the physician and the decision maker.	Review	N/A	Adult	Doctor/ parent interaction	SDM	Clinicians acknowledge their own cultural beliefs and values (including those stemming from the culture of medicine), maintain awareness of potential biases and assumptions, appreciate the complexity of patient and family identities and narratives, practice cultural humility, understand the moral relevance of culture, and respect patient and family preferences for SDM.
Michelson KN (2020) (18)	American Journal of Critical Care, 29;(4):271-282	To test the feasibility of studying and implementing a PICU communication intervention called PICU Supports.	Prospective/ Observation	53	Paediatrics	Doctor/ parent interaction	PICU	An interventionist trained in PICU-focused health care navigation, a "navigator," met with parents and the HCPs to discuss1) communication, 2) decision-making, 3) emotional, 4) informational, and 5) discharge or end-of-life care needs.

Odeniyi F (2017) (19)	Journal of Pain and Symptom Management, 54;(6):909-915	To describe experiences and challenges faced by paediatric oncologists and intensivists and how the oncologist-intensivist relationship impacts communication and initiation of goals of care discussions.	Qualitative study design/ Interviews	10	Adult	Doctor	End of life	Goal of care discussions with families of oncology patients in ICU. Three barriers included; 1) challenges to communication within teams because of hierarchy and between teams due to incomplete sharing of information and confusion about who should initiate GCDs; 2) provider experiences of internal conflict about how to engage parents in decision-making and about the "right thing to do" for patients; and 3) lack of education and training in communication. Four facilitators included 1) team preparation for family meetings; skills for partnering with families; 2) the presence of palliative care specialists; 3) informal education in communication and 4) willingness for further training in communication.
Ruhe KM (2016) (20)	European Journal of Pediatrics, 175;(9):1147-1155	To explore how to engage participation of children and adolescents in their healthcare.	Qualitative study design/ Interviews	52	Paediatric	Doctor/ parent/ Child	Communication	Three themes were identified; (a) modes of participation that captured the different ways in which children and adolescents were involved in their healthcare; (b) regulatory mechanisms that allowed children, parents, and oncologists to adapt patient involvement in communication and decision-making; and (c) other factors that influenced patient participation.
Gough JK (2009) (21)	Journal of Paediatrics & Child Health, 45;(3):133-8	The aim of our study was to obtain a better understanding of the trainees' experiences of a simulation programme in giving parents bad news.	Qualitative study design/ Interviews	9	Adult	Doctor	Communication	Five themes emerged from the qualitative data: 1) timeliness, 2) emotional safety, 3) the complexity of communication, 4) practical usefulness and 5) the challenge of effecting change.
Friedman J (2018) (22)	Acta Paediatrica, 107;(1):33-39	To characterise NICUstaff perceptions regarding factors which may lead to more challenging staff-parent interactions, and beneficial strategies for working with families with whom such interactions occur.	Questionnaires	270	Paediatrics	Parents	CMC	Strategies for improving difficult interations were: 1) frequent family meetings, 2) grieving opportunities, 3) education of parents, 4) social work referrals, 5) clearly defined rules, 6) partnering in daily care and 7) support groups.
Limacher R (2023) (23)	BMC Palliative Care, 22;(1):53	To investigate the communication patterns and contents between neonatal HCPs and parents of neonates with life-limiting or life-threatening conditions.	Qualitative study design Interviews	16	Paediatrics	Doctors/ Parents	Communication	Three main themes: 1) the weight of uncertainty in diagnosis and prognosis, 2) the decision-making process, and 3) palliative care.

Mitchell S (2019) (24)	BMJ Open, 9;(5):e028548	To provide an in-depth insight into the experience and perceptions of bereaved parents who have experienced end of life care decision-making for children with life-limiting or life-threatening conditions in PICU.	Qualitative study design/ Interviews	17	Paediatrics	Parents	End of life	Five interconnected themes were identified related to end of life care decision-making:(1) parents have significant knowledge and experiences that influence the decision-making process.(2) Trusted relationships with HCPs are key to supporting parents making end of life decisions.(3) Verbal and non-verbal communication with HCPs impacts on the family experience.(4) Engaging with end of life care decision-making can be emotionally overwhelming, but becomes possible if parents reach a 'place of acceptance' and 5) Families perceive benefits to receiving end of life care for their child in a PICU.
Myers J (2018) (25)	The American journal of Hospice & Palliative Care, 35;(8):1123-1132	To provide evidence regarding tools and/or practices available for use by healthcare providers to effectively facilitate advance care planning conversations and/or goals of care discussions.	Review	N/A	Adult	Doctors	End of life	Effective advance care planning conversations at both the population and the individual level require; 1) provider education and communication skill development, 2) standardized and accessible documentation, 3) quality improvement initiatives, and 4) system-wide coordination to impact the population level.
Aslakson RA (2014) (26)	Critical Care Medicine, 42;(11):2418-28	To review opportunities to improve palliative care for critically ill adults identifying resources to support implementation.	Review	N/A	Adult	Doctor	End of life	Four themes were identified, 1) opportunities to alleviate physical and emotional symptoms, improve communication, and provide support for patients and families; 2) models and specific interventions for improving ICU palliative care; 3) available resources for ICU palliative care improvement; and 4) ongoing challenges and targets for future research.
Bernacki R (2019) (27)	JAMA Internal Medicine, 179;(6):751-759	To examine feasibility, acceptability, and effect of a communication quality-improvement intervention (Serious Illness Care Program) on patient outcomes.	RCT	365	Adult	Doctor/ Patient	Communication	The coprimary outcomes included goal-concordant care (Life Priorities) and peacefulness (Peace, Equanimity, and Acceptance in the Cancer Experience questionnaire) at the end of life. Secondary outcomes included therapeutic alliance (Human Connection Scale), anxiety (Generalized Anxiety Disorder 7 scale), depression (Patient Health Questionnaire 9), and survival. Uptake and effectiveness of clinician training, clinician use of the conversation tool, and conversation duration were evaluated. Significant reductions in anxiety and depression in the intervention group.
Seccareccia D (2015) (28)	Journal of Palliative Medicine, 18;(9):758-64	To identify elements of communication that are central to quality of care and satisfaction with care on palliative care units.	Qualitative study design	46	Adult	Doctor/patients	Communication	Communication was the most prevalent theme regarding satisfaction and quality of care, with five subthemes describing elements important to patients, caregivers, and staff. These included: 1) building rapport with patients and families to build trust and kinship; 2) addressing expectations and explaining goals of care; 3) keeping patients and families informed about the patient's condition; 4) listening actively to validate patients' concerns and individual needs; and 5) providing a safe space for conversations about death and dying.

Boyd C (2019) (29)	Journal of the American Geriatrics Society, 67;(4):665-673	To translate principles into a framework of Actions and accompanying Action Steps for decision making for clinicians who provide both primary and specialty care to older people with multiple chronic conditions	Review	N/A	Adult	Doctor	CMC	Recommendations include, (1) identify and communicate patients' health priorities and health trajectory; (2) stop, start, or continue care based on health priorities, potential benefit vs harm and burden, and health trajectory; and (3) align decisions and care among patients, caregivers, and other clinicians with patients' health priorities and health trajectory.
Sisk BA (2022) (30)	Pediatric Blood & Cancer, 69;(10):1	To identify how to engage adolescents and young adults (AYAs) with cancer in communication and care.	Qualitative study design/ Interviews	37	Paediatric	Children	Communication	Additionally, we identified five factors that influenced adolescent and young adults' roles in communication and care, 1) AYA agency, 2) clinician encouragement, 3) emotional and physical well-being, 4) personality, preferences, and values, and 5) insights and skills.
Olsson MM (2021) (31)	Journal of Pain & Symptom Management, 62;(2):425-437.e2	To explore existing guidelines around palliative care to increase current understanding of end-of-life communication processes applicable to the acute care setting.	Review	N/A	Adult	Doctor	Communication	Eight themes were identified, 1) The purpose and process of end-of-life communications, 2) cognitive understanding and language in end-of-life communication, 3) legal aspects of end-of-life communication, 4) conflicts and barriers related to end-of-life care, 5) end-of-life communication related to medical record documentation, 6) HCPs ' responsibilities and collaboration, 7) education and training, and 8) policies, guidelines, and tools for end-of-life communications.
Sanders JJ (2018) (32)	Journal of Palliative Medicine; 21:S17-S27	To propose measurement priorities for serious illness communication and its anticipated outcomes, including goal-concordant care.	Review	N/A	Adult	Doctor	Communication	Implementation-ready measures to assess the quality of serious illness communication and care include, 1) the timing and setting of serious illness communication, 2) patient experience of communication and care, and 3) caregiver bereavement surveys that include assessment of perceived goal concordance of care. Future measurement priorities include direct assessment of communication quality, prospective patient or family assessment of care concordance with goals, and assessment of the bereaved caregiver experience.
Fine E (2010) (33)	Journal of Palliative Medicine, 13;(5):595-603	To review studies that used direct observation methods in palliative/end-of-life care communication research.	Review	N/A	Adult	Doctor	Communication	Four common themes were identified, 1) physicians focus on medical/technical and avoid emotional/quality of life issues; 2) sensitive topics are perceived by physicians to take longer to discuss and often do take longer to discuss; 3) physicians dominate discussions; and 4) patient/family satisfaction is associated with supportive physician behaviours.
Garcia-Quintero X (2022) (34)	Children, 9;6	To complete the Essential Messages in Palliative Care and Pain Management in Children (EmPalPed), an educational toolkit to increase awareness and promote essential knowledge in palliative care and pain management for low- and middle-income countries.	Prospective study/ Questionnaires	145	Paediatric	Doctor	Education	The toolkit had five key domains; 1) PC as it relates to the concept of quality of life (QoL), 2) effective communication, 3) addressing pain management as a top priority, 4) providing end-of-life care, and 5) access to high-quality PC as a fundamental human right. The workshop activities included different educational strategies and tools (e.g., a pocket guide for pain assessment and management, a PPC booklet, a quick guide for communicating bad news, role playing, and discussions of clinical cases).

Van Scoy LJ (2016) (35)	Journal of Pain & Symptom Management, 52;(5):655-662	To evaluate an electronic web-based tool which assembles the patient, their caregivers, and their healthcare providers in a virtual space for team-based communication.	Qualitative study design/ Interviews	18	Adult	Doctor/ Patient	Communication	Five themes relating to electronic communication to their experience of care, 1) apparent gaps in care, 2) uncertainty in defining the circle of care, 3) relational aspects of communication, 4) incongruence between technology and social norms of patient-physician communication, and 5) appreciation but apprehension about the team-based communication tool for improving the experience of care.
Heath G (2016) (36)	Healthcare, 4;1	To develop a tool to involve parents in their child's hospital care.	Qualitative study design/ Interviews	24	Paediatric	Doctor/ parents	Communication	"Listening To You" communications bundle, including a survey, literature review and consultation with parents and staff. Communication bundle implemented associated with paediatric early warning scores (PEWS) and escalation of care.
Kwame A (2021) (37)	BMC Nurs, 3;20(1):158	To identify the barriers and facilitators of patient-centered care and communication and propose and present a patient-centered care and communication continuum (PC4) Model to explain how patient-centered care can be enhanced in nurse-patient clinical interactions.	Review	N/A	Adult	HCP	Communication	A person-centred care and communication continuum (PC4 Model) is proposed to orient HCPs to care practices, discourse contexts, and communication contents and forms that can enhance or impede the achievement of patient-centred care in clinical practice.
Neubauer K (2018) (38)	Cardiology in the Young, 28:1088-1092	To summarise what is known about parent preference for communication and decision making in children with congenital heart disease (CHD).	Review	N/A	Paediatric (CHD)	Parents	Communication	Three themes regarding physician-parent communication and decision-making in the context of paediatric heart disease; 1) amount, timing, and content of information provided to parents; 2) helpful physician characteristics and communication styles; and 3) reinforcing the support circle for families.
Wreesmaan W (2021) (39)	Patient Edu & Counselling, 104; (7): 1505-1517	To assess the main functions of parent-provider communication in the NICU and determine what adequate communication entails according to both parents and HCPs.	Review	N/A	Paediatric	HCPs/ Parents	Communication	Four themes around the function of communication; 1) building/maintaining relationships, 2) exchanging information, 3) (sharing) decision-making, 4) enabling parent self-management) and five factors that contribute to adequate communication across these functions (topic, aims, location, route, design) and, thereby, to tailored parent-provider communication.
Labrie N (2021) (40)	Patient Edu & Counselling, 104; (7): 1526-1552	To synthesize and analyse the literature on the effects of parent-provider communication during infant hospitalization in NICU on parent-related outcomes.	Review	N/A	Paediatric	HCPs/ Parents	Communication	Five (positive and negative) effects of parent-provider interaction on parents', 1) coping, 2) knowledge, 3) participation, 4) parenting, and 5) satisfaction. Communication interventions appeared impactful, particularly in reducing parental stress and anxiety. Findings confirm and refine the NICU Communication Framework.

McSherry M (2022) (41)	Crit Care Med 1-14	To summarise the literature on prognostic, goals-of-care conversations in PICU.	Review	N/A	Paediatric	HCPs/ Parents	Communication	Future research should identify evidence-based communication practices that enhance caregiver-clinician prognostic and goals of care communication in PICU should include; 1) caregiver and clinician perspectives of underserved and limited English proficiency populations, 2) inclusion of caregivers who are not physically present at the bedside, 3) standardized communication training programs with broader multidisciplinary staff inclusion, 4) improved design of patient and caregiver educational materials, 5) the development of paediatric decision aids, and 6) inclusion of long-term post-PICU outcomes as a measure for PGOCC interventions.
Foster M (2013) (42)	J of Family Nurs 19;(4):431-468	To explore the attitudes, experiences, and implementation of family centred care.	Review	N/A	Paediatric	HCPs/ Parents	Communication	Nine themes emerged; 1) prehospital, 2) entry into hospital, 3) journeying through unknown waters, 4) information, 5) relationships, 6) the hospital environment, 7) the possibility of death, 8) religion and 9) spirituality.
Salmon P (2019) (43)	Patient Edu & Counselling, 102; (7): 1401-1403	To identify communication skills training that could improve patient outcomes	Review	N/A	Paediatric	HCPs/ Parents	Communication	Communication skills training need to include the following within research to evaluate how patients benefit from these initiatives, 1) choosing the primary outcomes, 2) a theoretical framework clear meaning to specific outcomes, 3) deciding on a target level of change, 4) the sample size and 5) what kind of research is needed to answer uncertainties
Baenzinger J (2020) (44)	Support Cancer Care 28; 4467-4476	To understand and address parents difficulties when interacting with HCPs.	Qualitative study design/ interviews	58	Paediatric	Parents	Communication	Parents' experiences were characterized by, 1) positive and negative interactions, 2) attitudes towards health care and HCPs, 3) trust and mistrust in the doctor-parent relationship, and 4) parents' engagement in care.
Hallman M (2020) (45)	Critical Care Nurse 40(2):e1-12	To assess communication in paediatric critical care from the provider, parent, and patient perspective.	Review	N/A	Paediatric	HCPs/ Parents/ Children	Communication	Findings indicate that effective communication is challenging in PICU despite robust evidence that effective communication improves patient outcomes and quality metrics. Repeated and varied forms of communication, especially written reinforced with verbal communication, seem to have the strongest effect.
Harbaugh B (2004) (46)	Comprehensive Pediatric Nursing 27;(3):163-178	To understand the parent perceptions of PICU hospitalization may help nurses with addressing the need to humanize the experience.	Qualitative study design/ interviews	19	Paediatric	Parents	Communication	Parents reported nurses engaged in nurturing and vigilant behaviour, namely showing affection, caring, watching, and protecting. Parents' reports suggest that the best nursing behaviours are those that facilitate and complement critical aspects of the parental role, thus reinforcing family integrity during a time of turmoil and uncertainty.
Nobile C (2003) (47)	Developmental & Behavioural Paed	To review research on parent-provider communication in paediatrics.	Review	N/A	Paediatric	HCPs/ Parents	Communication	This review recommends the development of more effective studies of parent-provider communication and relevant interventions in paediatric primary care include the need for theoretical models to help guide research, the development of reliable and valid self-report measures of communication, the assessment of clinically relevant correlates of parent-provider communication, and the study of children's roles in communication with their paediatricians.

Boland L (2019) (48)	Implementation Science 14:7	To synthesize barriers and facilitators from the perspective of HCPs, parents, children and observers.	Review	N/A	Paediatric	HCPs/ Parents	Shared decision making (SDM)	The most frequent barriers were features of the options (decision), poor quality information (innovation), parent/child emotional state (adopter), power relations (relational), and insufficient time (environment). The most frequent facilitators were low stake decisions (decision), good quality information (innovation), agreement with SDM (adopter), trust and respect (relational), and SDM tools/resources (environment).
Feudtner C (2007) (49)	Pediatri Clin North Am 54(4):583	To provide a framework for collaborative communication between patients, families, and clinician.	Review	N/A	Paediatric	HCPs/ Parents	Communication	Collaborative communication comprises of five important tasks; 1) establishing a common goal or set of goals that guide our collaborative efforts; 2) exhibiting mutual respect and compassion for each other; 3) developing a sufficiently complete understanding of our differing perspectives; 4) assuring maximum clarity and correctness of what we communicate to each other and 5) managing intra-personal and interpersonal processes that affect how we send, receive, and process information
Thode M (2020) (50)	BMJ Supportive & Palliative Care 0;1-8	To provide an overview of the feasibility and effectiveness of tools that support communication between HCPs and patients regarding decisions on life-prolonging treatments in hospital settings.	Review	N/A	Adults	HCPs/ Parents	End of life	Five studies described question prompt lists, either as a stand-alone tool or as part of a multifaceted programme. Question prompt lists were considered feasible by both patients with advanced cancer and HCPs. Question prompt lists can lead to a decrease in patient anxiety and an increase in cues for discussing end-of-life care with physicians.
Graetz D (2022) (51)	Cancer 15;128(10):1888-1893	To introduce a functional communication framework that can be used for global paediatric cancer research.	Review	N/A	Paediatrics	HCPs	Cancer	Supporting communication requires a framework that is adaptable to local circumstances and culture.

Supplementary File S3 Table S3. Parental communication survey response.

Questions	Numbers	Min	Max	Mean	SD
**Survey Means**	**29**	**1**	**5**	**4.06**	**0.15**
**Sharing information …..**How often do your child's doctors and other health professionals...					
Use words that you can understand?	29	2	5	4.28	0.84
Give you as much information as you want?	29	3	5	4.55	0.63
Show interest in what you think about your child's condition?	29	2	5	4.52	0.78
Ask if you understand the information they give you?	29	2	5	4.17	1.04
Doctor	29	2	5	4.03	1.02
Nurses	29	4	5	4.79	0.41
Dietitians	29	2	5	4.11	0.88
Other HCPs	29	2	5	3.97	0.91
Help you to understand complicated information?	29	2	5	4.31	0.71
Seem to understand what you tell them about your child's experience?	29	3	5	4.21	0.77
Make sure that you understand their explanations?	29	1	5	4.07	1.07
**Relationships with Doctors and Other Health Professionals**					
Admit when they do not know something?	29	1	5	3.41	1.15
Put your child's needs above everything else?	29	2	5	4.46	0.79
Show that they care about you?	29	2	5	4.21	0.94
Show that they care about your child as a person, and not just their illness?	29	2	5	4.31	0.93
Seem to be experts in treating your child?	29	3	5	4.48	0.69
Respond quickly to new medical problems?	29	3	5	4.59	0.68
Spend as much time with you as you need?	29	2	5	4.10	0.98
Seem informed and up-to date about your child's medical care?	29	2	5	4.28	0.84
**Making Decisions**					
Give you all the information you need to make decisions?	29	2	5	4.28	0.92
Give you options in your child's medical care?	29	2	5	4.11	0.99
Show interest in what you think about medical decisions?	29	2	5	4.10	0.90
Explain the different options you have?	29	2	5	4.07	0.92
Explain what they recommend?	29	3	5	4.24	0.83
**Dealing with Uncertainty**					
Help you make decisions based on what you think is important?	29	2	5	3.97	0.91
Involve you in making decisions as much as you want?	29	2	5	3.97	0.98
Help you deal with uncertainties about your child?	29	2	5	3.90	1.05
Help you to know what to expect before starting procedures?	29	2	5	4.21	0.90
Talk about what might happen to your child's health in the future?	29	2	5	3.59	1.02
Discuss things that are uncertain about your child's progress?	29	2	5	3.90	0.86
Help you to understand what is likely to happen your child?	29	3	5	4.07	0.70
Seem to understand what worries you about your child's health in the future?	29	2	5	4.10	0.82
**Taking care of your child**					
Make sure you can follow the treatment plan?	29	3	5	4.17	0.71
Take your needs into account when scheduling visits?	29	2	5	4.04	0.92
Tell you about helpful resources and support services?	29	2	5	3.97	1.05
Help you to get everything you need to care for your child?	29	2	5	4.17	0.85
Talk with you about ways you can manage your child's side effects to medication or symptoms?	29	1	5	3.69	1.11
Tell you how to deal with problems that come up in your child's care?	29	2	5	3.83	0.89
Make sure you understand the plan for your child's care?	29	2	5	4.00	1.00

Teach you everything you need to know to care for your child?	29	2	5	3.83	0.97
**Attention to Your Emotions**					
Ask how you are doing emotionally?	29	2	5	3.90	1.01
Give the right amount of attention to your emotions?	29	2	5	3.93	1.07
Show concern about how you are doing emotionally?	29	2	5	3.76	0.95
Show concern for how your child is doing emotionally?	29	1	5	4.10	1.01
Make you feel comfortable talking about your fears, stresses, and other feelings?	29	1	5	3.83	1.20
Give resources to help you cope with your fears, stresses, and other feelings?	29	1	5	3.66	1.20
Support you in the way you want?	29	2	5	4.00	0.89
**Providing Validation**					
Make you feel listened to and understood?	29	2	5	4.07	0.84
Value your thoughts about your child's health?	29	2	5	4.14	0.83
Take your concerns seriously?	29	2	5	4.17	0.85
Encourage you to speak up when you are concerned about your child?	29	2	5	4.28	0.96
Doctors/HCPs	29	2	5	4.10	0.86
Encourage you to ask questions?	29	2	5	4.34	0.86
Remind you that you are a good parent?	29	1	5	3.66	1.04
Make you feel like an important part of the team?	29	1	5	3.83	1.10
Doctors/HCPs	29	1	5	3.45	1.27
**Supporting hope**					
Help you to feel hopeful?	29	2	5	3.72	1.07
Help you to set goals that are important for you?	29	2	5	3.59	1.05
Help you to find things to hope for about your child's treatment/ hospital stay	29	2	5	3.83	1.04
Drs / HCPs	29	2	5	4.17	0.97

*highlighted cells with mean scores below the survey mean

Supplementary File S4. Chloe's card - What do you think about the tool?

This survey is estimated to take four minutes to complete and has been designed to get a better understanding of what you think about Chloe's card This survey asks questions about use of Chloe's card as a parent or health care professional.

It is completely anonymous.

If you are happy to continue with this survey, just click the button below, or simply close the survey if you do not want to take part

◉ Yes, I am happy to proceed to the survey.

About you…………………

Where are you?

E1 Ocean ward	
PICU	
Neonates	

Are you a...

Health care professional	
Parent	

Health care professional

**As a HCP did you find Chloe's card in your day to day work……**	Yes	No
Easy to use?		
Was useful in helping to start a conversation?		
Was useful in helping to reduce parental worries?		
Was useful in helping to build a relationship with parents?		
Was burdensome and something else I need to do?		
Did you feel Chloe's card is useful?		
Do you think it would benefit future parents?		

As a parent did you find Chloe's card...

**As a HCP did you find Chloe's card in your day to day work……**	Yes	No
Easy to use?		
Needed very little explanation		
Useful in helping to start a conversation		
Helped you to tell the team important information about your child		
Helped reduce worries		
Helped to build a relationship with the medical team		
Did you feel Chloe's card is useful?		
Do you think it would benefit future parents?		

Your views on Chloe's card

What has worked well using Chloe's card?

What didn't work so well using Chloe's card?

Is there anything we should change about Chloe's card?

